# Neuromodulation of Glial Function During Neurodegeneration

**DOI:** 10.3389/fncel.2020.00278

**Published:** 2020-08-21

**Authors:** Rebecca Stevenson, Evgeniia Samokhina, Ilaria Rossetti, John W. Morley, Yossi Buskila

**Affiliations:** ^1^School of Medicine, Western Sydney University, Campbelltown, NSW, Australia; ^2^International Centre for Neuromorphic Systems, The MARCS Institute for Brain, Behaviour and Development, Penrith, NSW, Australia

**Keywords:** glia, neurodegeneration, astrocytes, spatial buffering, K^+^ clearance

## Abstract

Glia, a non-excitable cell type once considered merely as the connective tissue between neurons, is nowadays acknowledged for its essential contribution to multiple physiological processes including learning, memory formation, excitability, synaptic plasticity, ion homeostasis, and energy metabolism. Moreover, as glia are key players in the brain immune system and provide structural and nutritional support for neurons, they are intimately involved in multiple neurological disorders. Recent advances have demonstrated that glial cells, specifically microglia and astroglia, are involved in several neurodegenerative diseases including Amyotrophic lateral sclerosis (ALS), Epilepsy, Parkinson’s disease (PD), Alzheimer’s disease (AD), and frontotemporal dementia (FTD). While there is compelling evidence for glial modulation of synaptic formation and regulation that affect neuronal signal processing and activity, in this manuscript we will review recent findings on neuronal activity that affect glial function, specifically during neurodegenerative disorders. We will discuss the nature of each glial malfunction, its specificity to each disorder, overall contribution to the disease progression and assess its potential as a future therapeutic target.

## Introduction

Glia are non-neuronal cells of the nervous system which do not generate electrical impulses yet communicate *via* other means such as calcium signals. Due to their lack of electrical activity, it was previously assumed that glial cells primarily functioned as “nerve-glue” (Virchow, 1860) and performed house-keeping functions for neurons; however, this concept has shifted due to recent findings showing glia are key components in many neuronal functions that go far beyond housekeeping (Araque et al., 1999; Buskila et al., 2019a).

Glial cells are categorized into two main groups; macroglia, which includes astrocytes, oligodendrocytes, NG2-glia and ependymal cells, and microglia which are the resident phagocytes of the central nervous system (CNS). Each population of glial cells is specialized for a particular function in the central or peripheral nervous system (García-Cabezas et al., 2016), and normal brain function depends on the interplay between neurons and the various types of glial cells. In this review, we will focus on astrocytes and microglia.

### Main Processes Involving Glial Cells

Glia are involved in many fundamental processes in the CNS including regulation of microcirculation (Zonta et al., 2003), formation and pruning of synapses (Allen et al., 2012), synaptic plasticity (Buskila and Amitai, 2010), and learning and memory (Ben Menachem-Zidon et al., 2011). Glia are also essential for the regulation of the blood-brain barrier (BBB; Lippmann et al., 2012), clearance of metabolic waste during sleep (Xie et al., 2013) and maintenance of synaptic transmission *via* ion homeostasis which includes K^+^ clearance (Capuani et al., 2016) and uptake of neurotransmitters (Lehre and Danbolt, 1998). Moreover, glia plays a key role during neuroinflammation (Kékesi et al., 2019) and their activity determines its severity and overall neurotoxic effects (Liddelow et al., 2017).

### Synapse Formation, Maintenance, and Pruning

Glia play a key role in synapse formation and function during development and adulthood (John Lin et al., 2017). Synapse development can be divided into synaptogenesis, pruning, and stabilization (Koeppen et al., 2018). Microglia and astroglia are ideally positioned to affect these activities as they are in close association with synapses, axons, and dendrites, which allows them to monitor and alter synaptic functions (Perez-Alvarez et al., 2014). Evidence suggests synapses can be formed without glial support (Ullian et al., 2001); however, these synapses are functionally premature. Hence, interactions between neurons and glia are essential for synapse generation and function (Ullian et al., 2004; Stogsdill and Eroglu, 2017).

During synaptogenesis, astrocytes play a pivotal role in the formation and regulation of the number of synapses. This was first recognized in retinal ganglion cells cultured in the absence of astroglia, which formed 10-fold fewer synapses compared to cultures containing astrocytes (Ullian et al., 2001). Moreover, astrocyte-conditioned medium originated from astrocytes from different brain regions, including the midbrain, cortex, hippocampus, and cerebellum, found to differ in their gene expression profiles, which effect their synaptogenesis potential (Buosi et al., 2018). The formation of synapses requires the assembly of presynaptic axon terminals and postsynaptic dendritic processes into a functioning unit that allows the transmission of electrochemical signals (Priller et al., 2006; Zeng et al., 2018). This transformation is induced by astrocytic secreted factors including EphrinA3 (Iadecola and Nedergaard, 2007; Nishida and Okabe, 2007; Schiweck et al., 2018), thrombospondin (Christopherson et al., 2005; Xu et al., 2010), neuroligin (Stogsdill and Eroglu, 2017), and D-Serine (Sultan et al., 2015) which are involved in the formation of excitatory synapses. Moreover, astrocytes are involved in the regulation of molecules that can modulate synaptic plasticity and long-term potentiation (Ben Menachem-Zidon et al., 2011), including glutamate, ATP, adenosine and lactate (Araque et al., 1998; Coco et al., 2003; Martín et al., 2007; Sotelo-Hitschfeld et al., 2015; Lepannetier et al., 2018; Gonçalves et al., 2019). Astrocytes also seem to regulate the stability of synapses *via* direct contact, as they are essential for the morphological maturation of dendritic protrusions and promote their conversion into spines (Nishida and Okabe, 2007).

In addition to their role during synaptogenesis, plasticity, and maintenance, glial cells can remove synapses in a process termed synaptic pruning, further supporting their role in memory and learning processes (Jo et al., 2014). Microglial processes are constantly scavenging the extracellular space for pathogens and cellular debris. Throughout this surveillance process, in which they are in contact with axons, synapses, and dendritic spines, they phagocyte disused synapses (Lauterbach and Klein, 2006; Paolicelli et al., 2011; Hong et al., 2016b; Diaz-Aparicio et al., 2020; Milior et al., 2020), and therefore prevent energy and nutrient loss that is much needed at more active synapses. Hence, this synaptic pruning optimizes the effectiveness of neuronal transmission and functional plasticity in the brain and has been postulated to improve learning and memory capability (Millán et al., 2019). Indeed, abnormalities in synaptic pruning by microglia have been associated with the cognitive decline that accompanies many neurodegenerative disorders such as Alzheimer’s disease (AD; Hong et al., 2016a).

Astrocytes have also been implicated in synapse loss and regulation of long-term memory (Ben Menachem-Zidon et al., 2011). A recent study showed that over-expression of the protein Ephrin-B1 in astrocytes (that is typically linked to synapse formation and stabilization) led to synaptic loss and a decrease of glutamatergic signals (Koeppen et al., 2018). It is thought that during physiological activity, Ephrin-B1 expressed in astrocytes regulates synaptic remodeling by competing with neuronal Ephrin-B1 and thus restricting new synapse formation (Nikolakopoulou et al., 2016; Koeppen et al., 2018). Moreover, Nikolakopoulou et al. (2016) suggested that astrocytic Ephrin-B1 is activated *via* the astrocytic STAT-3 mediated signaling and affects EphB receptors in microglia which then triggers microglia-mediated synaptic pruning. These recent studies provide new insights about astrocyte-microglia communication that can impact synaptic pruning and therefore should be investigated as a new therapeutic target to prevent synapse loss in neurodegenerative diseases.

### Ion Homeostasis

A crucial glial function is the maintenance of the concentration of ions in the interstitial space to allow proper synaptic transmission (Capuani et al., 2016). Neuronal activity causes a transitory rise in extracellular K^+^ concentration, which must be cleared to avoid hyperactivity of the neuron (Chever et al., 2010). K^+^ clearance takes place primarily by active transport through astrocytic transporters and channels (termed K^+^ uptake) which then transport K^+^ *via* gap junctions to other astrocytes and back to the extracellular space, in a process known as spatial buffering (Orkand et al., 1966; Buskila et al., 2019a). This is an essential astrocytic function that prevents the harmful build-up of K^+^ levels in the extracellular space (Bellot-Saez et al., 2017). Impairments in astrocytic K^+^ clearance are thought to contribute to hypersynchronous oscillations (Bellot-Saez et al., 2018) and initiation of seizure activity in various neurological disorders as recently reviewed by Bellot-Saez et al. (2017).

In addition to K^+^, glial cells are responsible for the regulation of several other ions in the extracellular space including sodium (*via* the activity of the Na^+^, K^+^-ATPase together with a plethora of voltage-gated sodium channels; Wang et al., 2012), chloride (Ratté and Prescott, 2011) and hydrogen (*via* the activity of the Na^+^-HCO_3_ and Na^+^/H^+^ exchanger (Ghandour et al., 2000). Moreover, glia form the glymphatic system, which is responsible for the removal of metabolic waste products in the brain, facilitated by Aquaporin-4 (AQP4) channels expressed in astrocytic end-feet (Iliff et al., 2012). Astrocytic AQP4 channels facilitate CSF-ISF exchange and thus modulate water flux (and shrinkage) from the astrocyte to optimize the interstitial space during neuronal activity (Nagelhus et al., 2004) and the sleep-wake cycle (Ding et al., 2016).

### Uptake of Neurotransmitters

For proper synaptic transmission, neurotransmitters (e.g., GABA, glycine, and glutamate) must be transported away from the synaptic cleft immediately after release. Glutamate is the most common excitatory neurotransmitter in the brain, however, excess glutamate residing in the extracellular space can be neurotoxic (Ye et al., 2013). Many cells in the CNS are involved in glutamate reuptake, but astrocytes are responsible for the majority of this uptake (Bergles and Jahr, 1997; Lutgen et al., 2016) with some estimates as high as 60–80% of total glutamate uptake (Rothstein et al., 1996) *via* the excitatory amino acid transporters EAAT1 and EAAT2 (Bergles and Jahr, 1998). Hence, changes in astrocytic function to regulate neurotransmitter uptake are prone to affect synaptic function as well as cytotoxicity. Indeed, glutamate excitotoxicity is implicated in some neurodegenerative diseases, for a detailed review see Dong et al. (2009).

### Neurovascular Coupling and the BBB

The BBB is a highly selective border around the cerebral vasculature that prevents pathogens, toxic substances, red blood cells, and leukocytes from the circulating blood to cross into the interstitial fluid (Kanmogne et al., 2007). Astrocytes form a crucial part of the BBB by supporting the integrity of the tight junctions between endothelial cells and facilitating the provision of nutrients from blood vessels (Lippmann et al., 2012). Moreover, astrocytes regulate the constriction and dilation of microvessels (Iadecola and Nedergaard, 2007) and thus form the bridge in the neurovascular unit that couples the activity of neurons, astrocytes and BBB endothelial cells (Viggars et al., 2011). These features allow astrocytes to regulate the cerebral blood flow (CBF) in regions of high neuronal activity that requires more glucose and oxygen (Logothetis et al., 2001; Metea and Newman, 2006).

Previous studies indicated a close association between aging and the breakdown of the BBB, suggesting that impairment of neurovascular coupling is responsible for the cognitive decline seen in the elderly (Fabiani et al., 2014; Balbi et al., 2015) and during neurodegenerative diseases such as AD (Fabiani et al., 2014; Tarantini et al., 2017) and MS (Sivakolundu et al., 2020).

### Metabolic Waste and the Glymphatic System

The brain has a high metabolic rate (Attwell and Laughlin, 2001), yet does not have a traditional lymphatic system to clear waste products; instead, the glymphatic system, a term coined by the Nedergaard group (Xie et al., 2013), is utilized as the functional waste clearance system of the CNS. This glial-dependant system facilitates the removal of metabolic by-products and soluble proteins from the brain parenchyma *via* the exchange of solutes between the interstitial fluid and the cerebrospinal fluid (Xie et al., 2013; Wang et al., 2017). The exchange of fluids is driven by arterial pulsation and activity of AQP4 channels expressed in astrocytic endfeet (Iliff et al., 2012), suggesting that impairment of the BBB function can impact waste clearance mechanisms, induction of neuroinflammation and thus neurodegeneration, as seen in BBB associated pathology (Yang et al., 2013). Indeed, AQP4 knockout mice experienced a 55–65% reduction in Aβ clearance compared to WT mice (Iliff et al., 2012). Furthermore, a 40% reduction has been reported in aged mice compared to young mice (Kress et al., 2014), which is consistent with the notion that the glymphatic system is compromised with age, while age is also the biggest risk factor for dementia and several neurodegenerative conditions such as in late-onset AD (Alzheimer’s disease facts and figures; Alzheimer’s Association, 2019). Sleep has also been shown to affect the glymphatic system, with the clearance of toxic proteins during sleep occurring twice as fast as during awake periods in mice (Xie et al., 2013).

### Neuroinflammation

Neuroinflammation is an immune response to harmful stimuli in the nervous system, triggered by infection or injury that leads to microglia and astrocyte activation, aiming to defend the neuronal tissue by clearing and controlling the harmful stimuli (Davalos et al., 2005). Microglia are the resident immune cells of the CNS and are known to actively investigate their microenvironment using their processes (Nimmerjahn et al., 2005). During an injury, infection, or disease state, microglia become “activated,” whereby they migrate towards sites of injury or infection and initiate an inflammatory response by producing cytokines and chemokines (Brockhaus et al., 1996). However, recent studies showed that microglial activation can span between the canonical pro-inflammatory response (termed M1 microglia) to an alternative activation (termed M2), in which microglia release anti-inflammatory cytokines (e.g., BDNF, IGF-1, and TGF-β), and facilitate phagocytosis, tissue repair, and regeneration (Park et al., 2016; Zhou et al., 2017). Moreover, the M2 phenotype is associated with increased Aβ clearance in AD (Kawahara et al., 2012), neurogenesis and higher survival rate of progenitor stem cells (Yuan et al., 2017), indicating that shifting microglia into the M2 phenotype is a plausible treatment in neurodegenerative disorders, as recently suggested by Zhang et al. (2018).

However, some have argued the M1/M2 polarization model is a conceptual deficit and needs to be reconsidered as the categorization into “classical” or “alternative” state is an over simplification that does not capture the heterogeneity of microglia (for extensive reviews see Martinez and Gordon, 2014; Ransohoff, 2016). Indeed, transcriptomics has uncovered gene expression changes much beyond what can be appreciated with a handful of markers. One such study looked at gene expression profiles of mice that underwent traumatic brain injury (TBI). Individual cells were analyzed using RNA-sequencing to investigate if M1-like or M2-like macrophages could be detected (Kim et al., 2016). The study found that expression of a polarization marker was no more likely than random to be co-expressed within the same cell, concluding that microglia are not made up of polarized groups, but proposing that individual macrophages undergo a wide range of activation (Kim et al., 2016).

Like microglia, astrocytic activation is also hypothesized to range between two poles of a spectrum. On the one side of the spectrum, A1 activated astrocytes promote cell death, the release of neurotoxins (i.e., IL-1β, IL-6, TNF-α, IFN-γ) and activation of catabolic processes (Liddelow et al., 2017). On the other side of the spectrum, A2 astrocytes are beneficial as they provide neuroprotection by releasing antioxidants and neurotrophic factors *via* activation of the JAK/STAT3 pathway (Zamanian et al., 2012; Liddelow et al., 2017). Evidence suggests that A1 reactive astrocytes are induced by cytokines released from activated microglia (i.e., Il-1α, TNFα, and C1q; Zhang et al., 2014). These activated A1 astrocytes lose the capacity to support neuronal survival, induce the death of oligodendrocytes and neurons and accompany many neurodegenerative diseases such as Huntington’s disease (HD), AD and Amyotrophic lateral sclerosis (ALS; Liddelow et al., 2017). Hence, the prevention of A1 astrocytic formation and transformation of A1 astrocytes towards the A2 phenotype can serve as a therapeutic target. Although this classification provides insight towards astrocytic heterogeneity, the categorization of activated astrocytes into the A1/A2 model has been recently challenged and concerns have been raised that this classification would be used as a shortcut to imply a mechanistic understanding (Vainchtein and Molofsky, 2020). Cunningham et al. (2019) recently suggested that this classification is oversimplified which puts into risk the complexity of the factors shaping astrocyte phenotype, and thus astrocytic heterogeneity. Evidence in the field is yet to establish whether the A1/A2 classification is suitable, with recent opinion questioning if the grouping may be a simplification of the various cellular states that astroglia assumes, both in diseased and healthy states (Arranz and De Strooper, 2019).

## Neuron to Glia Communication Pathways

### Neurotransmission *via* the Tripartite Synapse

The tripartite synapse is a functional unit comprised of a neuronal component (pre- and post-synaptic membranes) along with an ensheathing astrocyte (Araque et al., 1999). Evidence shows that the number of tripartite synapses varies between brain regions, suggesting different levels of neuron-glial communication. In the rodent brain, synapses ensheathed by astrocytes account for 60–90% of total synapses in the cerebellum (Xu-Friedman et al., 2001), 77% in the striatum (Chai et al., 2017), 86% in the hippocampus (Chai et al., 2017) and more than 90% of synapses in the barrel cortex (Genoud et al., 2006). Although it remains unclear if tripartite synapse numbers in rodents are comparable to humans, a previous study shows that human astrocytes are significantly larger and structurally more complex when compared to rodents (Oberheim et al., 2009). However, this number may be harder to estimate as tripartite synapses are dynamic and can change following neuronal activity or sensory stimulation (Schipke et al., 2008). It is not well documented how these synapses and their associated mechanisms are affected in pathology, however, evidence suggests that an imbalance in the tripartite synapse activity is implicated in epilepsy and neuropsychiatric disorders such as schizophrenia (Mitterauer, 2018).

### Amplification of Neuronal Signaling *via* Neuromodulation of Glial Activity

Astrocytes are functionally coupled *via* gap junctions comprised of a pair of connexons which connect across intracellular space (Nagy et al., 2001; Johnson et al., 2018) and link the cytoplasm of two cells (Hofer and Dermietzel, 1998; Wei et al., 2004; Wilson and Mongin, 2019). Astrocytic gap junctions have several functions, including facilitation of electrical and chemical communication between cells (Kettenmann and Ranson, 1988), affecting neuronal rhythmicity in the brainstem (Kadala et al., 2015; Condamine et al., 2018), distribution of excess K^+^ from high concentration regions to low concentration areas, and allowing transmembrane transport of small molecules to share metabolic demands across cells (Huguet et al., 2016). This communication method allows for astrocytes to work as a syncytium and propagate signals through the astrocytic network (Halassa et al., 2007). The failure of such connectivity and dysfunction of signal propagation results in a malfunctioning nervous system and can be a factor in the progression of neurodegenerative disorders.

Astrocytic connectivity is also paramount for neuronal—astrocytic communication. Previous studies showed that neuronal activity triggers glial responses, resulting in changes in the astrocytic Ca^2+^ concentration (Poskanzer and Yuste, 2011, 2016). Astrocytes respond to neurotransmitters and neuromodulators *via* activation of G-protein coupled receptors (GPCRs) which leads to the release of 1,4,5-triphosphate that induces Ca^2+^ release (Lev-Ram and Ellisman, 1995). These glial Ca^2+^ signals are not restricted to a single cell and can propagate as a calcium wave within the glial syncytium (Cornell-Bell et al., 1990; Schipke et al., 2002) and thus amplify the original neuronal signal, as recently proposed by Chen et al. (2019).

**Figure 1:**
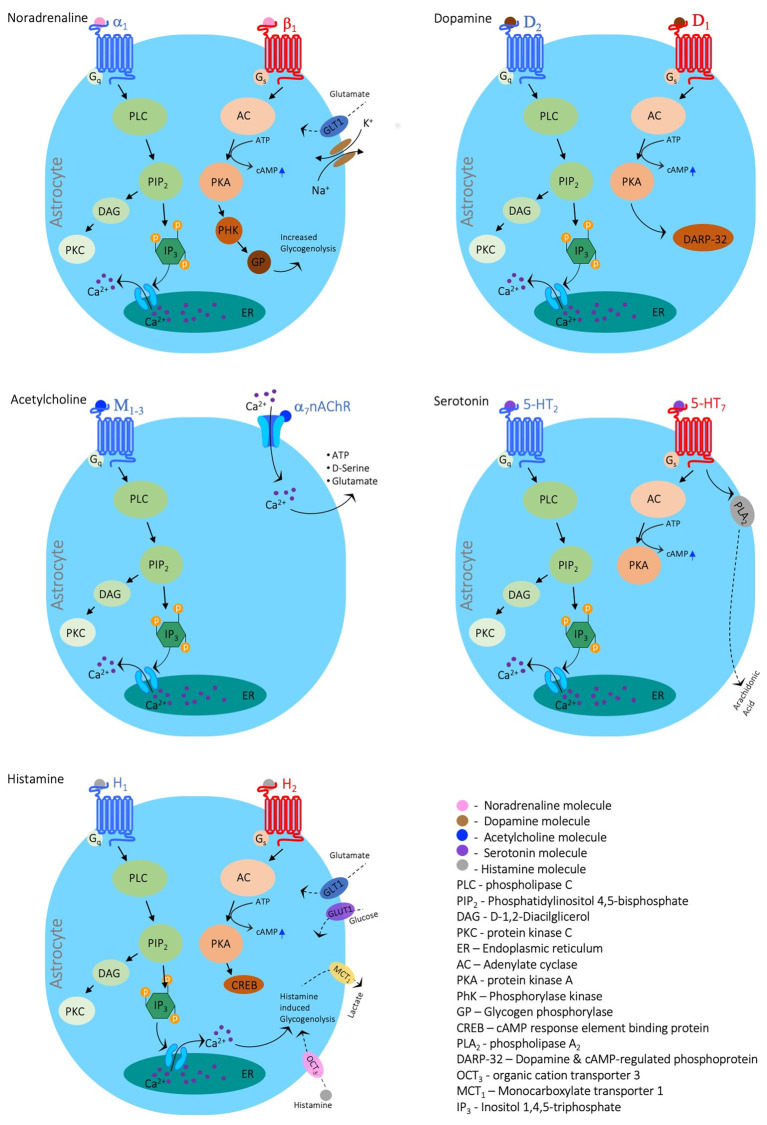
Neuromodulators’ impact on astrocytic calcium signaling. Schematic diagrams depicting the main signaling cascades in which the neuromodulators Noradrenaline, Dopamine, Acetylcholine, Serotonin, and Histamine evoke calcium signals. The figure was created with the Motifolio toolkit (Motifolio Inc., Ellicott City, MD, USA).

Neurons can modulate glial function *via* activation of a wide variety of receptors for different neuromodulators, including acetylcholine (Ach, nicotinic α/β and metabotropic M_1–4_; Oikawa et al., 2005; Amar et al., 2010), histamine (H_1–3_; Inagaki et al., 1989), serotonin (5-HT_1,2,5,6,7_; Hirst et al., 1998), Noradrenaline (NA, α_1,2_-adrenoreceptors and α_1,2_-adrenoreceptors; Bekar et al., 2008; Morin et al., 1997) and dopamine (DA, D_1–5_; Khan et al., 2001; Wei et al., 2018). In most cases, the neuromodulatory agent will lead to either change in calcium signaling in astrocytes (see Figure 1) and microglia or alterations of the coupling ratio, affecting glia to work as a syncitium. Indeed, recently, Ma et al. (2016) showed that neuromodulators can signal through astrocytes by affecting their Ca^2+^ oscillations to alter neuronal circuitry and consequently behavioral output. In line with these observations, Nedergaard’s group further demonstrated that bath application of a cocktail of neuromodulators to cortical brain slices increased [K^+^]_o_ regardless of synaptic activity (Ding et al., 2016), suggesting that increased [K^+^]_o_ could serve as a mechanism to maximize the impact of neuromodulators on synchronous activity and recruitment of neurons into networks (Bellot-Saez et al., 2018; Zupanc, 2020). Moreover, Bellot-Saez et al. (2019) recently showed that different neuromodulators, such as 5-HT, NA, DA, and histamine affect the astrocytic K^+^ clearance rate *via* different pathways, establishing a synergistic mechanism between neurons and astrocytes that promote excitability and network oscillations. Also, Chen et al. (2019) reported that in the hypothalamus, norepinephrine activates corticotropin-releasing hormone (CRH) neurons to engage astrocytes *via* dendritic retrograde signaling. This activation leads to astrocytic release of ATP in upstream neurons, thus allowing CRH neurons to amplify their dendritic signaling and control distal presynaptic neurons.

Both noradrenaline and serotonin exhibit profound effects on astrocytic metabolism (Cambray-Deakin et al., 1988). Adrenergic agonists cause a disruption in astrocytic metabolism in the form of increased ATPase activity (Kimelberg et al., 1978) and have been shown to prime astrocytes for local circuity alterations and changes in behavioral state (Paukert et al., 2014). Whereas serotonin has the effect of altering K^+^ permeability in glial cells of rodent astrocytes (Hösli et al., 1993). It is hypothesized that a breakdown of communication in the adrenergic pathway in the locus coeruleus and the serotonergic pathway in the raphe nuclei leads to a break down in metabolic and functional neuron-glia communication causing the degradation of neurons in those regions, contributing to Alzheimer’s progression (Hertz, 1989).

However, the impact of neuromodulators on glial activity is complex and highly dependent on the receptor identity. For example, activation of α7 nAChRs led to a decrease in the release of pro-inflammatory cytokines such as IL-6 and TNF-α (Liu et al., 2012; Li et al., 2013; Kalashnyk et al., 2014), an increased expression of glial cell-derived neurotrophic factor (GDNF; Takarada et al., 2012) and upregulation of glutamate uptake *via* the glutamate/aspartate transporter (GLAST; Mashimo et al., 2010; Morioka et al., 2014), suggesting acetylcholine promotes neuroprotection *via* inhibition of microglia. On the other hand, ACh was found to cause an increase in intracellular Ca^2+^ which stimulates the release of glutamate (Wang et al., 2013), triggering GABAergic transmission in astrocytes (Banerjee et al., 2012; Beggiato et al., 2013) and thus affecting the activity of network oscillations. Moreover, cholinergic transmission affects satellite glia to enwrap and support sensory peripheral neurons that were previously deprived of nerve growth factor (Enes et al., 2020).

Recently, Bosson et al. (2015) showed that blockade of dopaminergic transmission in the substantia nigra increased astrocytic calcium activity and gap junction coupling, resembling morphological alterations of Parkinson’s disease (PD; Bosson et al., 2015), and further indicating that neuron-glia dopaminergic communication may play a part in the etiology of PD. While histamine plays a critical role in maintaining the energy balance of the CNS by regulating glucose allocation/distribution (Jurič et al., 2011), it is also purported to increase the excitability of neurons and increase astrocytic uptake of Ca^2+^ *in vitro* (Jung et al., 2000). Further exploration of the effect of neuromodulators on astrocytic processes will enrich our understanding of astrocyte functionality and the roles they play in healthy and pathological states.

### Neuronal Signals That Affect Neuroinflammation

While our understanding of the neuroinflammatory process is constantly developing it is clear that it is a common characteristic which is preserved across several neurodegenerative diseases (Thameem Dheen et al., 2007) including PD (Ghadery et al., 2017), AD (Schuitemaker et al., 2013), ALS (Sargsyan et al., 2005) and multiple sclerosis (MS; Kozela et al., 2011). A plausible explanation for this phenotype is that the inflammatory profile of microglia changes with age (DiSabato et al., 2016). It is thought that microglia in the aged brain is primed, as demonstrated by their elevated expression of inflammatory mediators, with a reduced activation threshold and higher levels of inflammation following activation. Moreover, once activated, aged microglia are more resistant to regulation (Norden and Godbout, 2013), which can be a contributing factor to the progression of neurodegenerative diseases, as active microglia synthesize and release neurotoxic substances that cause further neuronal damage (Lull and Block, 2010). In a healthy environment, activation of microglia serves as a protective mechanism, however, due to the complex and multifactorial nature of microglial activation, subtle changes may lead to amplified or dysfunctional activation which is thought to enhance neurodegenerative pathology, with cytokines presenting at almost all disease phases (Brambilla, 2019).

Previous studies showed that glial activation is controlled by neuronal activity, which can “turn-on” or “turn-off” signals that either keep microglia in their resting quiescent state or activate them into a proinflammatory state, for a detailed review see Biber et al. (2007). One of the main agents through which neurons affect glial activation is the CD200 glycoprotein, which inhibits microglial priming and holds microglia in a quiescent state (Shrivastava et al., 2012). Indeed, alterations in the distribution of the CD200 or its glial receptor CD200R can lead to overactivation of microglia and neuroinflammation (Hernangómez et al., 2012) that accompany neurodegeneration (Oria et al., 2018). Other neuronal agents that affect the course of neuroinflammation are chemokines, such as CX3CL1, CXCR4 and CCL2 which promotes microglial scavenging (Meucci et al., 2000; Hatori et al., 2002; Paolicelli et al., 2014), regulation of the BBB integrity (Rabinovich-Nikitin et al., 2016) and chemotaxis (Coughlan et al., 2000; Conductier et al., 2010) respectively. These chemokines activate diverse intracellular signaling pathways, including PI3K, AKT, and NF-κB to regulate phagocytosis, proliferation, and migration.

The Janus kinase/signal transducer and activator of transcription 3 (JAK/STAT3) pathway is a common mediator of astrocyte reactivity in neurodegenerative diseases (ND) and is highly conserved between disease states (Ben Haim et al., 2015). Previous studies showed that this pathway shifts astrocytes towards the more beneficial A2 phenotype, which reduces inflammation and astrogliosis (Okada et al., 2006). Indeed, recently Ben Haim et al. (2015) showed that inhibition of the (JAK/STAT3) pathway increased the number of pathological huntingtin aggregates in animal models for HD. On the other hand, activation of the NF-κB pathway in glial cells during inflammation was found to promote neurotoxicity and neurodegeneration, reviewed by Dresselhaus and Meffert (2019).

Some molecules released by neurons are found to have a dual effect on the development and progression of neuroinflammation. For example, the brain-derived neurotrophic factor (BDNF), which is released from neurons, can either activate the JAK/STAT3 pathway, which shifts microglia and astrocytes towards a more beneficial phenotype that reduces inflammation and astrogliosis (Okada et al., 2006; Lund et al., 2008; Hixson et al., 2019), or activate the receptor TrkB which leads to activation of the NF-κB detrimental pathways (Colombo et al., 2012). These alterations might stem from the mode of activation, as continuous administration of BDNF or its overexpression led to attenuation of microglial activation in a mouse model of HD (Lund et al., 2008; Xie et al., 2010; Giampà et al., 2013; Hixson et al., 2019), while acute administration of BDNF increased microglial activity (Jiang et al., 2010) and accelerated neurodegeneration (Colombo et al., 2012).

Glial recruitment and activation during neuroinflammation are also modulated by neuronal release of neurotransmitters (e.g., glutamate and GABA) and extracellular nucleotides, in particular ATP and UDP (Haynes et al., 2006; Ulmann et al., 2013). While GABA and glycine lead to a decrease in the LPS-induced secretion of several cytokines (Kuhn et al., 2004) and attenuation of the phagocytic activity of microglia (Carmans et al., 2010), the neuronal release of glutamate can either inhibit microglia activation *via* metabotropic receptors (Taylor et al., 2002, 2003) or mediate glial activation *via* AMPA and kainate ionotropic receptors (Hagino et al., 2004). Moreover, the release mechanisms of ATP (non-specific release as occurs during necrosis vs. vesicular exocytosis), were found to be key effectors on the inflammatory pathway and are capable of fine-tuning the glial response throughout neuroinflammation, reviewed by Dosch et al. (2018). Given the importance of the above signaling pathways on the progression of inflammation, in which, even minor changes are sufficient to cause fatal dysregulation of glial function and neurodegeneration, they are increasingly becoming the focus in the development of new therapeutics.

## Glial Processes Which Are Affected During Neurodegeneration

**Figure 2:**
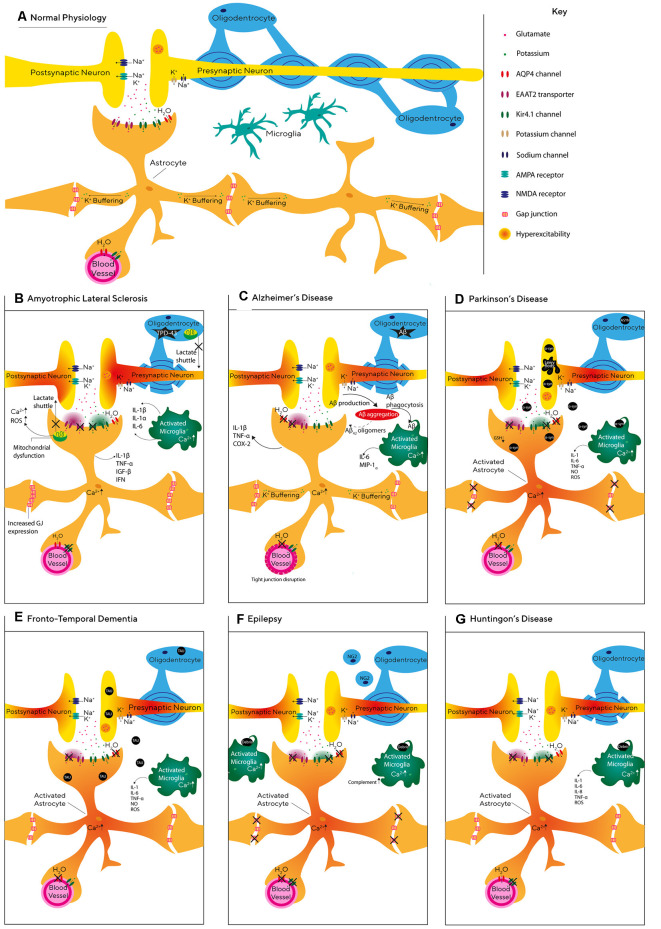
Glial pathophysiology during neurodegeneration. **(A)** Schematic representation of the relationship between neurons and glial cells, including astrocytes, oligodendrocytes, and microglia. **(B–G)** Graphic illustration of the molecular and cellular processes affecting astrocytes, microglia, and oligodendrocytes during Alzheimer’s disease (AD; **C**), Epilepsy **(F)**, Frontotemporal dementia (FTD; **E**), Parkinson’s disease (PD; **D**), Huntington disease **(G)**, and amyotrophic lateral sclerosis (ALS; **B**).

Neurodegenerative diseases are conventionally regarded as a progressive neurological disorder associated with the loss of specific populations of neurons. As a result, neurodegenerative diseases such as Parkinson disease, AD, epilepsy, and ALS have traditionally been associated with neurodegeneration of dopaminergic, cholinergic, GABAergic and motor neurons respectively (Davies and Maloney, 1976; Leenders et al., 1990; Xu-Friedman et al., 2001; Buskila et al., 2019b). However, increasing evidence suggests that glial dysfunction may be a significant contributor to the pathology of these diseases, as indicated in Supplementary Table S1. In this chapter, we will summarize specific contributions of glial dysfunction to different neurodegenerative diseases (see also Figure 2).

### Epilepsy

Epilepsy is a complex neurodegenerative disorder characterized by recurrent seizures and includes both focal and generalized seizures (Devinsky et al., 2013). Epilepsy can develop for several reasons including genetic disposition (Ottman, 2005), TBI, infections (Löscher et al., 2015), prenatal injury (Scher, 2003), and developmental disorders (Lehéricy et al., 1995). Seizures are thought to be caused by the synchronized firing of groups of neurons which results from an imbalance between inhibitory and excitatory currents (Scharfman, 2007). Therefore, previous epilepsy research focused on neurons, however, recent evidence indicates that glial cells are also involved in the pathology of epilepsy.

One of the hallmarks of acquired epilepsy is glial scarring formed by hypertrophic astrocytes (Das et al., 2012; Do Nascimento et al., 2012), in which the gap-junction connectivity between astrocytes is reduced (Wallraff et al., 2006). Indeed, recently Bedner et al. (2015) showed that impairments in astrocytic gap-junction coupling start within 4 h following status epilepticus. In line with this study, acute brain slices prepared from transgenic mice with coupling-deficient astrocytes expressed spontaneous epileptiform activity and increased synaptic transmission (Wallraff et al., 2006; Pannasch et al., 2011). Dysfunctional astrocytes may be pivotal to the generation and propagation of epileptic seizures (Seifert et al., 2006) *via* their inability to sufficiently clear K^+^, as increasing extracellular K^+^ concentration has been shown to increase synchronous oscillatory activity (Bellot-Saez et al., 2018) and induce epileptiform activity in brain slices (Gabriel et al., 2004). K^+^ buffering in astrocytes is also regulated by Kir4.1 channels which are found in astrocytic endfeet (Zurolo et al., 2012). Indeed, the variation of the KCNJ10 gene that encodes Kir4.1 channels has been linked to seizure susceptibility (Ferraro et al., 2004; Wallraff et al., 2006). Moreover, dysfunctional astrocytes with decreased expression of Kir4.1 channels displayed impaired clearance of extracellular K^+^ and glutamate which led to neuronal hyperexcitability (Patel et al., 2019). Although there is an ongoing debate as to whether gliosis is consequential or a causative effect of seizures (Patel et al., 2019), evidence in support of the latter has shown that astrogliosis alone can cause spontaneous seizures in the absence of other pathologies (Robel et al., 2015).

It has been hypothesized for some time that astrocytic processes grow towards synapses which release high levels of glutamate as a mechanism to offset the spill-over and prevent hyperexcitability (Ventura and Harris, 1999). Indeed, previous studies found that patients with epilepsy expressed lower levels of glutamine synthetase (GS) in the hippocampus (Petroff et al., 2002; Eid et al., 2004), which results in accumulation of glutamate in astrocytes and the extracellular space. Similarly, animal models for epilepsy showed a significant reduction of GS activity during the chronic epileptogenic phase (Kang et al., 2006; Bidmon et al., 2008; Hammer et al., 2008). Hence, it has been suggested that reduction in GS activity is the main reason for increased extracellular glutamate levels and seizure generation.

Research has established that astrocytes alter their morphology and domain organization in neurodegenerative disease, to the extent that there is a 10-fold increase in overlap of astrocytic processes in mouse models of epilepsy (Golarai et al., 2008). A recent study suggested that during the progression of epilepsy, the tripartite synapse undergoes molecular, structural, and functional changes in the hippocampus (Clarkson et al., 2020). The study reported that in addition to decreased density in GLT-1 expression, astrocyte processes increased ensheathment at synapses in an attempt to decrease excitability by raising the levels of glutamate uptake (Clarkson et al., 2020). Another astrocytic mechanism that can lead to epilepsy involves the dislocation of AQP4 water channels and reduced expression of Kir channels in astrocytes, which affect K^+^ clearance and enhance the propagation of seizures (Lee et al., 2004; Binder et al., 2006; Seifert et al., 2006; Szu et al., 2020) These findings demonstrate the importance of neuron-glia communication mechanisms when considering new therapeutic targets for epilepsy.

### Huntington’s Disease (HD)

HD is an inherited brain disorder characterized by the progressive loss of selected neuronal populations within the corticostriatal circuit and glial cell malfunction (Tong et al., 2014; Jiang et al., 2017). One of the earliest astrocytic dysfunctions in HD is a decrease in the expression of glutamate transporters (Faideau et al., 2010) and a decrease in extracellular glutamate uptake in both the striatum and cerebral cortex (Przedborski et al., 2000; Liévens et al., 2001; Behrens et al., 2002; Shin et al., 2005; Hassel et al., 2008; Baljit et al., 2017). Moreover, the mutated huntingtin protein is reported to lead to a decrease in the expression of Kir4.1 channels and impairment of astrocytic K^+^ clearance capabilities (Tong et al., 2014), leading to hyperexcitability of local striatal neurons (Tong et al., 2014; Benraiss et al., 2016). Indeed, restoring the expression of astrocytic Kir4.1 channels in an HD mouse model rescued glutamate signaling, emphasizing the importance of K^+^ homeostasis in the development of HD (Jiang et al., 2016).

Recently, the Khakh group reported that astrocyte morphology and spatial territory at the striatum are significantly lower and precede synaptic dysfunction in an HD mouse model (Octeau et al., 2018). Dysfunctional glial cells have been shown to play a critical role in HD progression, as mutant huntingtin expressing human glial progenitor cells engrafted in healthy mice were found to impart the disease phenotypes (Benraiss et al., 2016). Moreover, the introduction of healthy human glial cells into transgenic HD mice significantly increased survival rates and reduced motor deterioration, along with the reinstatement of K^+^ homeostasis (Benraiss et al., 2016). Furthermore, Garcia et al. (2019) used patient-derived HD astrocytes to demonstrate that the dysfunction of these cells leads to less astrocytic support and decreased protection from glutamate toxicity, suggesting astrocytic malfunction should be regarded as a major therapeutic target in HD.

### Amyotrophic Lateral Sclerosis (ALS)

ALS is the most common type of motor neuron disease, affecting the brain and spinal cord and causing progressive loss of the upper and lower motor neurons controlling muscle movement, breathing, and speech (Martin et al., 2017). Astrocytes are thought to contribute to the progression of ALS through the abnormal function of glutamate transporters and extracellular K^+^ concentration, leading to elevated levels of extracellular glutamate and excitotoxicity (Rothstein et al., 1995; Do-Ha et al., 2018). Indeed, SOD1 mutations that are thought to facilitate oxidative stress (Barber et al., 2006) and are typically found in patients who suffer from the familial form of ALS (Tang et al., 2019) were accompanied with reduced expression of EAAT2 transporters in astrocytes (Pedersen et al., 1998). Moreover, Gong et al. (2000) showed that the expression of mutated SOD1 must exist in both neurons and astrocytes for ALS to progress, and expression of mutated SOD1 in neurons alone was not sufficient to trigger ALS (Pramatarova et al., 2001). Although, other *in vivo* studies do indicate that transplanted glial-restricted precursor cells in wild-type rats are sufficient to induce motor neuron death (Papadeas et al., 2011). This is evidence that ALS is not a disease of astrocytes or neurons alone, and that both cell types are involved in the pathogenesis of ALS (Rothstein et al., 1995; Do-Ha et al., 2018).

### Frontotemporal Dementia

Frontotemporal dementia (FTD) is the most common type of dementia for those aged 65 and under (Hogan et al., 2016) and is characterized by atrophy of the frontal anterior temporal lobes (Broe et al., 2004). Although the role astrocytes play in FTD is not fully understood, a meta-analysis of FTD cases revealed that astrocytic cell death increased as the disease progressed, while neuronal apoptosis remained constant (Broe et al., 2004). These studies indicate that the neurodegeneration seen in FTD may be facilitated by the loss of astrocytic support. Indeed, a recent study conducted by Hallmann et al. (2017) showed that FTD astrocytes demonstrated changes in whole-genome expression including upregulation of genes linked with synapse organization and transmission (CHRNA1). Moreover, decreased expression of EAAT2 has also been observed in the frontal cortex of FTD patients compared with controls (Umoh et al., 2018), suggesting that deficiencies in astrocytic glutamate uptake may contribute to excitotoxicity and neuronal degeneration (see Figure 2).

FTD progression is accompanied by protein alterations, neuroinflammation, and vascular dysfunction (Martinac et al., 2001; Garbuzova-Davis et al., 2012), which profoundly involve microglia and astrocytes (Philips and Robberecht, 2011). Indeed, microglial activation has been associated with neuronal loss and TAU deposition suggesting that such microglial activation occurs in the early stages of the disease (Schofield et al., 2003). Moreover, autopsy samples from FTD patients expressed high levels of reactive astrocytes showing signs of degradation which would likely contribute to neuronal cell death (Su et al., 2000).

One of the hallmarks in FTD patients is a reduction of the resting CBF in the frontal, temporal and parietal cortices (Dopper et al., 2016), which is correlated with astrocytic damage and astrogliosis (Martinac et al., 2001). As evidence suggests that altered cerebral perfusion may be an early biomarker of FTD and may even show distinct perfusion patterns (Hu et al., 2010), therapeutics aimed at maintaining cerebral circulation, to retain astrocytic and neuronal health, maybe a beneficial future target.

### Parkinson’s Disease

PD is the second most common neurodegenerative disease (de Lau and Breteler, 2006) with symptoms including resting tremor and bradykinesia (Salarian et al., 2007). PD is characterized by the loss of dopaminergic neurons in the substantia nigra pars compacta (SNpc) and the formation of α-syn deposits known as Lewy bodies (Kordower et al., 2013; Diniz et al., 2019). Recently, Bosson et al. (2015) reported an increase in the number of glutamatergic synapses, which was accompanied by alterations in astrocytic morphology in the SNr of a rat model to PD. Moreover, intracerebroventricular injection of α-syn increased the number of astrocytes, the density of excitatory synapses, and the levels of TGF-b1 in the striatum of injected animals (Diniz et al., 2019). Similarly, astrocytic spatial territories were found to be larger in the striatum of parkinsonian monkeys (Villalba and Smith, 2011), suggesting that astrocytic remodeling is a compensatory mechanism aiming to decrease the high extracellular glutamate levels and overall neuronal hyperactivity observed in PD.

Neurodegeneration of dopaminergic neurons has also been associated with alteration in the glial antioxidative system. Solano et al. (2008) showed that dysfunction of the PD-associated protein Parkin can lead to a decrease in the synthesis of the antioxidant glutathione (GSH) by astrocytes, underlying the lower levels of GSH found in PD patients (Baillet et al., 2010). Indeed, previous studies indicate that the prevention of oxidative stress protected dopaminergic neurons in an animal model of PD (Chen et al., 2009; Kitamura et al., 2011), further supporting the role of glial malfunction in the degeneration of dopaminergic neurons.

It is well established that glial cells of post-mortem PD patients show high levels of α-syn accumulation (Wakabayashi et al., 2000). Direct neuron-glia transmission plays an essential role in the progression of PD *via* the release of α-syn at axon terminal sites where the α-syn is taken-up into astrocytes, triggering an inflammatory response (Lee et al., 2010). It is generally thought that astrocytes remove α-syn from the synapse as a protective mechanism (Booth et al., 2017), however, a recent study postulates that astrocytes amplify PD pathology by accumulating and spreading α-syn to neighboring dopamine-producing neurons and thus intensifying neurodegeneration (di Domenico et al., 2019). Indeed, cultured neurons originated from healthy individuals were more susceptible to neuronal death once they were in contact with iPSC-derived astrocytes from a PD patient. However, when healthy astrocytes were cultured with PD patient neurons, α-syn did not accumulate and neuronal function was restored (di Domenico et al., 2019). These studies highlight the role that malfunctioning astrocytes play in the spread of α-syn, accumulating abnormal levels due to impaired protein degradation which may enhance the spread of PD in the brain. As the SNpc region has the lowest astrocyte to neuron ratio in the CNS (Damier et al., 1993), this may explain why dopaminergic neurons are susceptible to PD as they have less astrocytic support than neurons in other regions (de Majo et al., 2020).

### Alzheimer’s Disease

AD, the most prevalent form of dementia worldwide (Ferri et al., 2005), is a progressive neurodegenerative disorder characterized by the build-up of β-amyloid (Aβ) plaques, intracellular neurofibrillary tangles (NFTs) with hyper-phosphorylated tau and activated glial cells surrounding senile plaques (Morley et al., 2018). Aβ deposits and tau are primarily derived from neurons, however, the genes associated with the development of late-onset Alzheimer’s, including apolipoprotein (APOE), apolipoproteinJ (APOJ) and sortilin-related-receptor-1 (SORL) are mainly expressed by glial cells (Arranz and De Strooper, 2019), suggesting that both glia and neurons are involved in the pathogenesis of the disease. Moreover, a recent report by Habib et al. (2020) identified a population of disease-associated astrocytes in an AD mouse model, which appeared at early disease stages and increased with disease progression.

Aβ deposits in the brain lead to early loss of synapses and neurons (Buskila et al., 2013), abnormal activation of microglia, and oxidative stress (Leyns et al., 2019). Recently, Beth Stevens’ group reported that phagocytic microglia mediate synaptic loss in the early stages of AD *via* the complement system, indicating an abnormal activation of the synaptic pruning pathway (Hong et al., 2016b). Moreover, there is growing evidence that glutamatergic communication between neurons and astrocytes at the tripartite synapse is affected during AD progression, as Aβ leads to increased glutamate release from neurons and astrocytes (Pirttimaki et al., 2013; Talantova et al., 2013). Previous studies in an animal model for AD demonstrated that astrocytes in the entorhinal cortex, prefrontal cortex, and hippocampus undergo morphological and functional alterations. These changes include a decrease in the number of astrocytes expressing the enzyme glutamine synthase, atrophy, and a reduction in the surface area, volume, and complexity of astrocytic processes (Olabarria et al., 2011; Beauquis et al., 2013, 2014; Kulijewicz-Nawrot et al., 2013). Moreover, astrocytes at the pre-symptomatic AD phase exhibited a decrease in vesicle trafficking (Stenovec et al., 2016), as well as lower expression of AQP4 and GLT-1, which results in the disruption of water and glutamate homeostasis (Hoshino et al., 2017) and an increase of glutamate release from astrocytes, leading to hyperexcitability and cell death (Buskila et al., 2005; Pirttimaki et al., 2013; Talantova et al., 2013).

### Multiple Sclerosis

Multiple sclerosis (MS) is a disease in which the myelin sheath of axons degenerates, resulting in a wide range of symptoms and functional impairments including pain, muscle weakness, and tremors (Trapp et al., 1998). The canonical view of this disease progression is that CD4^+^ T-lymphocytes cross the BBB into the parenchyma which causes a cascade of several events leading to inflammatory lesion and demyelination (Laplaud et al., 2004; Bahbouhi et al., 2009). However, a secondary hypothesis states that the disease is neurally initiated and begins in the CNS, caused by metabolic changes affecting neurons and glia which leads to BBB breakdown (Chaudhuri and Behan, 2004). Although somewhat controversial, evidence supporting this theory suggests that neuronal loss and inflammation are present at the very early stages of MS (Filippi et al., 2003). If it is the case that MS begins in the CNS, neuroprotective interventions may be effective as future MS treatments. However, the autoimmune vs. CNS-derived theories of MS are still the source of an ongoing debate in the literature.

The classification of different neurodegenerative diseases is based on certain neurological symptoms expressed in each disorder, however, there is considerable clinical and pathological comorbidity across neurodegenerative disorders. Patients with FTD may present with ALS or parkinsonian syndromes and vice versa (Lomen-Hoerth et al., 2002; Padovani et al., 2007). Also, major risk factors that lead to glial malfunction, such as cardiovascular dysfunction, TBI, and stroke are common in different neurodegenerative diseases, suggesting that similarities between different disorders stem from common glial dysfunction.

## Energy Metabolism During Neurodegeneration

The brain has a disproportionate energy demand relative to its size. While it constitutes only 2% of the total body weight, it requires 20% of the oxygen and glucose metabolism (Sokoloff, 1991). Hence, regulation of energy metabolism is essential to maintain proper brain function, see review by Watts et al. (2018). In the adult brain, both neurons and glia use glucose as an energy substrate, however, a small portion of glucose molecules in the brain is stored as glycogen chains preferentially within astrocytes (Obel et al., 2012).

Glycogen storage in the adult brain is highly compartmentalized, with the highest levels in the hippocampus, striatum, and superficial layers of the cortex (Oe et al., 2016). Immunohistochemical studies showed that glycogen has a punctate distribution localized predominantly in astrocytic processes (Oe et al., 2016; Hirase et al., 2019). These studies further indicated an astrocytic heterogeneity, whereas some astrocytes were “glycogen-rich” while others found to be glycogen-poor. Further investigation of the functional compartmentalization of glycogen stores in astrocytes showed that glycogen granules are preferentially located in astrocytic processes around synapses and blood vessels, rather than randomly distributed in the astrocytic cytosol (Calì et al., 2016; Mohammed et al., 2018). This distribution suggests that astrocytic glycogen plays a role in synaptic processes.

Historically, astrocytic glycogen was considered to be an emergency fuel, which is mobilized only once there is discontinuation in the cerebral glucose supply. However, glycogen content in the CNS is low (8–12 μmol/g tissue in the rat brain; Cruz and Dienel, 2002), and can support brain function only for a few minutes under ischemic conditions (Swanson et al., 1989). Recent studies suggest that glycogen metabolism is associated with different brain functions including the regulation of awake-sleep cycles and high cognitive processes such as learning and memory consolidation (Kong et al., 2002; Gibbs et al., 2007; Gibbs and Hutchinson, 2012; Gibbs, 2016; Bellesi et al., 2018). Moreover, glycogen is a precursor for glutamate and glutamine synthesis (Sickmann et al., 2005; Gibbs et al., 2007) and is found to play a neuroprotective role, as it protects neurons from stress conditions including hypoglycemia or hypoxic stress (Obel et al., 2012; Öz et al., 2015; Adeva-Andany et al., 2016).

Glycogen metabolism in astrocytes is actively regulated by neuromodulators, which activates adenyl cyclase and increases intracellular calcium levels (Sorg and Magistretti, 1992; Cardinaux and Magistretti, 1996; Sickmann et al., 2009; Obel et al., 2012; Dinuzzo et al., 2013; Xu et al., 2014). Classical studies by Pierre Magistretti’s group found that neuromodulators such as vasoactive intestinal peptide (VIP), histamine and NA, induce glycogenolysis (the breakdown of glycogen) in astrocytes and lead to a local increase of ATP within the stimulated networks in a concentration-dependent manner (Magistretti et al., 1981; Magistretti and Schorderet, 1984; Magistretti and Morrison, 1988). In line with this work, *in vivo* studies involving photostimulation of the retina, confirmed that sensory stimulation is associated with glial glycogenolysis (Tsacopoulos et al., 1988; Poitry-Yamate and Tsacopoulos, 1991). Moreover, glycogenolysis was detected in the somatosensory cortex of awake rats immediately after sensory stimulation of the vibrissae, further supporting the impact of neuromodulators on energy metabolism (Poitry-Yamate and Tsacopoulos, 1991; Swanson et al., 1992; Dienel et al., 2007). In contrast, under conditions of low glutamatergic and monoaminergic activity (e.g., anesthesia; slow-wave sleep), glycogen levels increase (Folbergrová et al., 1970; Watanabe and Passonneau, 1973; Karnovsky et al., 1983; Morgenthaler et al., 2006; Bellesi et al., 2018), suggesting that glycogen is mostly used during intense neuronal activity. Consistent with this hypothesis, Gibbs and Hutchinson (2012) showed that NA affects glycogen turnover to enhance memory consolidation and glycogen-derived lactate was found to be central to higher cognitive function and memory formation (Newman et al., 2011; Suzuki et al., 2011).

However, the impact of neuromodulators on astrocytic glycogen metabolism is complex and highly dependent on the identity of the receptors. For example, while activation of the hippocampal β1-adrenergic receptor was found to induce glycogenolysis during the transition between the first and second phases of intermediate memory, after 30 min it caused α2-adrenoreceptors to induce glycogen synthesis (Gibbs et al., 2007; Hutchinson et al., 2011; Gibbs and Hutchinson, 2012). Similar to noradrenaline, vasoactive intestinal polypeptide (VIP) in addition to its glycogenolytic effect, was also found to elicit temporally-delayed resynthesis of glycogen (Sorg and Magistretti, 1991).

Given the impact of neuromodulators on glycogen metabolism, any impairment in their activity is primed to affect the energy metabolism in the brain, as seen in different neurodegenerative diseases. Indeed, noradrenergic dysfunction during Alzheimer’s disease (Mann et al., 1987) has been linked to disruptions in glycogen metabolism (Hertz, 1989; Marien et al., 2004; Weinshenker, 2008). Other studies showed that epileptic patients had increased levels of glycogen in the hippocampus, and sustained seizures led to the synthesis and accumulation of abnormal glycogen molecules that are resistant to degradation (Dalsgaard et al., 2007; Dinuzzo et al., 2015). Accumulation of glycogen was also found in the spinal cord of animal models for ALS (Li et al., 2019) and epilepsy (Rubio-Villena et al., 2018), which was due to deteriorated glycogenolysis.

Energy metabolism in astrocytes is not only modulated by neurons but also affects neuronal activity. Specifically, glycogen-derived energy supports the clearance of extracellular potassium and glutamate following neuronal activation (Choi et al., 2012). Following the astrocyte-neuron lactate shuttle hypothesis, glucose-derived lactate from astrocytes is transferred to neurons to maintain neuronal metabolism (Pellerin et al., 2007), including oxidative phosphorylation and restoration of a Na^+^ gradient following glutamatergic neurotransmission (Sickmann et al., 2005; Gibbs et al., 2007). Inhibition of astrocyte energy turnover induces deterioration of both neuronal energy metabolism and extracellular clearance, which can cause neuronal hyperexcitability (Kreisman and Smith, 1993; Xu et al., 2013; Kilic et al., 2018). Hence, an initial disturbance in neuron-astrocyte interactions may create a vicious cycle of disease development through positive feedback loops, resulting in augmented neuronal disfunction.

## Glia as a Therapeutic Target for Treating Neurodegeneration

Glial cells make up an integral part of the CNS architecture and are critical for the maintenance of healthy brain function. In recent years the field has shifted from viewing glial cells as passive “housekeeping” cells to becoming active participants in brain function and modulation of neuronal output. In addition to their functions in the healthy brain, a growing body of evidence suggests glia are implicated in the progression of neurodegeneration, either through the loss of normal function or the introduction of abnormal function (Sofroniew and Vinters, 2010). Moreover, there is cumulative experimental evidence suggesting that functional and genetic alterations in glia are driving disease pathogenesis, see De Strooper and Karran (2016) and Gleichman and Carmichael (2020) for detailed reviews. For these reasons, neuron-glia transmission can no longer be viewed as a passive method of communication and must be seen as an active and important signaling thread that joins many neurodegenerative disorders.

Current therapeutic strategies for treating neurodegeneration include targeting the ApoE4 signaling pathway, which is the greatest risk factor for AD and primarily expressed in glial cells, and nicotinamide adenine dinucleotide (NAD), which is thought to regulate inflammatory repair processes (Blacher et al., 2015; Roboon et al., 2019). Indeed, Sawmiller et al. (2019) reported that disrupting the ApoE signaling cascade ameliorated cerebral Aβ and tau pathologies, including neuronal apoptosis and synaptic loss in a mouse model of AD. Moreover, the deletion of CD38, the enzyme that hydrolyzes NAD and controls its bioavailability, was recently found to suppress glial activation and neuroinflammation in a mouse model for demyelination (Roboon et al., 2019). As levels of NAD reduce with age, this strategy represents a promising target for MS, AD, and other age-related neurodegenerative disorders.

Another therapeutic strategy includes astrocyte-targeted production of interleukin-6, which has been shown to reduce the activation of both astrocytes and microglia and delay demyelination of neurons in a mouse model of MS (Petković et al., 2016). Moreover, targeting the histaminergic system with anti-histamine proved as a strategy that can preserve myelin and initiate myelination in animal models of MS (Mei et al., 2014, 2016; Liu et al., 2016), and this has now been tested in a randomized control trial of 50 MS patients with promising results (Green et al., 2017).

Recently, strategies directed towards targeting the astrocytic glutamate transporter EAAT2 proved to be beneficial in alleviating the hyperexcitability symptoms associated with some neurodegenerative diseases and decrease the level of neurodegeneration in animal models of epilepsy (Kong et al., 2012, 2014; Goodrich et al., 2013; Zaitsev et al., 2019), ALS (Kong et al., 2014), PD (Chotibut et al., 2014; Hsu et al., 2015) and HD (Sari et al., 2010). Moreover, modulation of astrocytic energy metabolism *via* inhibition of lactate dehydrogenase (Sada et al., 2015; Rosati et al., 2018) or *via* ketonic diet (Valdebenito et al., 2016) had a neuroprotective effect in both animal models of PD (Shaafi et al., 2016), and patients with HD (Adanyeguh et al., 2015) and epilepsy (van Berkel et al., 2018). Also, treatments that targeted the suppression of the inflammatory NF-κB pathway by increasing the expression of neurotrophic growth factor IGF-1 (Hu et al., 2018), or decreasing microglial proliferation (Martínez-Muriana et al., 2016), postponed the onset and slowed down the progression of motoneuron death in a mouse model of ALS.

## Conclusion

The etiology of neurodegeneration is still unclear, however recent studies established that lack of glial support and dysregulation in neuron-glia interactions are central for disease initiation and progression. More research is needed to address key questions on neuronal-glia interactions, such as why do astrocytes contact some synapses and not others, and how does this communication change over developmental stages or with the progression of the disease?

We know neuron-glia interactions are dynamic, can be altered by neuronal activity, and can change throughout life (Bernardinelli et al., 2014). However, during neurodegeneration, alterations in neuron-glia interactions take place slowly and their source is largely unknown. While glial modulation of synaptic morphology and function has been heavily investigated in the past decade, the neuron-glia communication processes that affect glial function are still unclear. Molecular, cellular, and genetic approaches can be employed to investigate these communication mechanisms, as addressing this gap will provide novel targets for much needed therapeutic intervention.

## Author Contributions

All authors contributed to the article and approved the submitted version.

## Conflict of Interest

The authors declare that the research was conducted in the absence of any commercial or financial relationships that could be construed as a potential conflict of interest.
