# Supplementary material for: Neuromodulation of Glial Function During Neurodegeneration
Source: Front Cell Neurosci. 2020 Aug 21;14:278. doi: 10.3389/fncel.2020.00278 (PMC7473408; doi:10.3389/fncel.2020.00278)
Supplement: Supplementary file 1 [file Table_1.DOCX]

*Supplementary table 1 – glial processes affected during neurodegeneration*

| **Neuro. disorder** | **Glial processes affected** | | |
| --- | --- | --- | --- |
|  | **Astrocytes** | **Microglia** | **Oligodendrocytes/NG2** |
| Focal epilepsy | - Alterations in gene expression of KCNJ10 (Ferraro et al., 2004); GS (Eid et al., 2004) and GLT-1 (Clarkson et al., 2020) - Astrogliosis (Robel et al., 2015) - Decreased gap-junction connectivity (Wallraff et al., 2006) - Reduced clearance capacity for extracellular glutamate and potassium (David et al., 2009) - Dislocation of AQP4 channels due to a decreased expression of the dystrophin gene (Lee et al., 2004) | - Increased proliferation, induction of lysosomal genes and phagocytosis (Zhao et al., 2018a) - Activation of classical complement pathway (Aronica et al., 2007) | - Proliferation of NG2 cells (Wu et al., 2019) |
| Alzheimer’s Disease | - Morphological and functional changes (Jones et al., 2017) - Dysregulation of Ca^2+^ signalling (Oksanen et al., 2017) - Decreased vesicle trafficking (Stenovec et al., 2016) - Alteration in the expression of AQP4 (Wilcock et al., 2009) and GLT-1 (Zeng et al., 2007) - Increased release of pro-inflammatory cytokines (Oksanen et al., 2017) | - Increased activation (Zhao et al., 2018b) - Dysregulation of Ca^2+^ homeostasis (Brawek et al., 2014) - Impairment of Aβ uptake and clearance (Griciuc et al., 2013) - Increased release of pro-inflammatory cytokines (Zhao et al., 2018b) | - Demyelination (Behrendt et al., 2013) - Decrease in Oligodendrocyte numbers in post-mortem brain of AD patients (Behrendt et al., 2013) - Increased number of mature oligodendrocytes in 6 months old mice of 3×Tg-AD model (Behrendt et al., 2013; Desai et al., 2010) |
| Multiple sclerosis | - Gliosis (Haindl et al., 2019) | - Early activation (Buschmann et al., 2012) - Upregulated phagocytosis (Voß et al., 2012) - Increased production of pro-inflammatory cytokines (Voß et al., 2012) | - Morphological changes of NG2 cells, expressed as an increase in size, domain and arborization (Barriola et al., 2020) - Dysregulation of OPCs maturation and proliferation leading to depletion of oligodendrocytes (Mason et al., 2004) |
| **Neuro. disorder** | **Glial processes affected** | | |
|  | **Astrocytes** | **Microglia** | **Oligodendrocytes/NG2** |
| Amyotrophic lateral sclerosis | - Enhanced production of pro-inflammatory cytokines and neurotoxic factors (Johann et al., 2015) - Increased oxidative stress (Birger et al., 2019) - Downregulation of Kir4.1 K^+^ channel (Bataveljić et al., 2012) and glutamate transporters (Howland et al., 2002) - Impairment of the lactate efflux transfer (Ferraiuolo et al., 2011) | - Enhanced production of pro-inflammatory cytokines and neurotoxic factors (Di Giorgio et al., 2008) - Increased oxidative stress (Zhao et al., 2004) | - Enhanced proliferation of OPCs generating dysmorphic oligodendrocytes (Philips et al., 2013) - Deficiency in lactate transport leading to axonal damage and neuronal loss (Lee et al., 2012) |
| Parkinson’s Disease | - Alterations in astrocytic morphology (Bosson et al., 2015) - Decreased synthesis of the antioxidant GSH by astrocytes (Solano et al., 2008) - Uncontrolled uptake and removal of dopaminergic neuronal debris (Morales et al., 2017) - Accumulation of α-syn (Wakabayashi et al., 2000) | - Upregulation of pro-inflammatory cytokines and phagocytic activity (Lastres-Becker et al., 2012) | - Accumulation of α-syn (Wakabayashi et al., 2000) |
| Huntington Disease | - Downregulation of Kir4.1 K^+^ channel and reduced clearance capacity for extracellular potassium (Tong et al., 2014) - Downregulation of glutamate transporters and reduced glutamate clearance capacity (Faideau et al., 2010) - Morphological changes (Faideau et al., 2010) | - Early activation at presymptomatic stage and increased phagocytosis (Savage et al., 2020) - Production of ROS, pro-inflammatory cytokines and neurotoxic molecules (Politis et al., 2015) | - Demyelination and reduced expression of myelin genes in mature oligodendrocytes (Huang et al., 2015) |
| FTD | - Gliosis and increased apoptosis (Broe et al., 2004) - Decreased expression of EAAT2 and impairment of glutamate uptake (Umoh et al., 2018) - Expression of 3R and 4R-Tau inclusions (Zhukareva et al., 2002) | - Early activation mediated by Tau deposition (Schofield et al., 2003) - Increased intrathecal production of both pro- and anti-inflammatory cytokines (Sjögren et al., 2004) | - Expression of 4R-Tau inclusions (Zhukareva et al., 2002) |

**References**

Aronica, E., Boer, K., van Vliet, E. A., Redeker, S., Baayen, J. C., Spliet, W. G. M., et al. (2007). Complement activation in experimental and human temporal lobe epilepsy. *Neurobiol. Dis.* 26, 497–511. doi:10.1016/j.nbd.2007.01.015.

Barriola, S., Pérez-Cerdá, F., Matute, C., Bribián, A., and López-Mascaraque, L. (2020). A Clonal NG2-Glia Cell Response in a Mouse Model of Multiple Sclerosis. *Cells* 9. doi:10.3390/cells9051279.

Bataveljić, D., Nikolić, L., Milosević, M., Todorović, N., and Andjus, P. R. (2012). Changes in the astrocytic aquaporin-4 and inwardly rectifying potassium channel expression in the brain of the amyotrophic lateral sclerosis SOD1G93A rat model. *Glia* 60, 1991–2003. doi:10.1002/glia.22414.

Behrendt, G., Baer, K., Buffo, A., Curtis, M. A., Faull, R. L., Rees, M. I., et al. (2013). Dynamic changes in myelin aberrations and oligodendrocyte generation in chronic amyloidosis in mice and men. *Glia* 61, 273–286. doi:10.1002/glia.22432.

Birger, A., Ben-Dor, I., Ottolenghi, M., Turetsky, T., Gil, Y., Sweetat, S., et al. (2019). Human iPSC-derived astrocytes from ALS patients with mutated C9ORF72 show increased oxidative stress and neurotoxicity. *EBioMedicine* 50, 274–289. doi:10.1016/j.ebiom.2019.11.026.

Bosson, A., Boisseau, S., Buisson, A., Savasta, M., and Albrieux, M. (2015). Disruption of dopaminergic transmission remodels tripartite synapse morphology and astrocytic calcium activity within substantia nigra pars reticulata. *Glia* 63, 673–683. doi:10.1002/glia.22777.

Brawek, B., Schwendele, B., Riester, K., Kohsaka, S., Lerdkrai, C., Liang, Y., et al. (2014). Impairment of in vivo calcium signaling in amyloid plaque-associated microglia. *Acta Neuropathol.* 127, 495–505. doi:10.1007/s00401-013-1242-2.

Broe, M., Kril, J., and Halliday, G. M. (2004). Astrocytic degeneration relates to the severity of disease in frontotemporal dementia. *Brain* 127, 2214–2220. doi:10.1093/brain/awh250.

Buschmann, J. P., Berger, K., Awad, H., Clarner, T., Beyer, C., and Kipp, M. (2012). Inflammatory response and chemokine expression in the white matter corpus callosum and gray matter cortex region during cuprizone-induced demyelination. *J. Mol. Neurosci.* 48, 66–76. doi:10.1007/s12031-012-9773-x.

Clarkson, C., Smeal, R. M., Hasenoehrl, M. G., White, J. A., Rubio, M. E., and Wilcox, K. S. (2020). Ultrastructural and functional changes at the tripartite synapse during epileptogenesis in a model of temporal lobe epilepsy. *Exp. Neurol.* 326, 113196. doi:10.1016/j.expneurol.2020.113196.

David, Y., Cacheaux, L. P., Ivens, S., Lapilover, E., Heinemann, U., Kaufer, D., et al. (2009). Astrocytic dysfunction in epileptogenesis: Consequence of altered potassium and glutamate homeostasis? *J. Neurosci.* 29, 10588–99. doi:10.1523/JNEUROSCI.2323-09.2009.

Desai, M. K., Mastrangelo, M. A., Ryan, D. A., Sudol, K. L., Narrow, W. C., and Bowers, W. J. (2010). Early oligodendrocyte/myelin pathology in Alzheimer’s disease mice constitutes a novel therapeutic target. *Am. J. Pathol.* 177, 1422–1435. doi:10.2353/ajpath.2010.100087.

Di Giorgio, F. P., Boulting, G. L., Bobrowicz, S., and Eggan, K. C. (2008). Human Embryonic Stem Cell-Derived Motor Neurons Are Sensitive to the Toxic Effect of Glial Cells Carrying an ALS-Causing Mutation. *Cell Stem Cell* 3, 637–648. doi:10.1016/j.stem.2008.09.017.

Eid, T., Thomas, M. J., Spencer, D. D., Rundén-Pran, E., Lai, J. C. K., Malthankar, G. V., et al. (2004). Loss of glutamine synthetase in the human epileptogenic hippocampus: Possible mechanism for raised extracellular glutamate in mesial temporal lobe epilepsy. *Lancet* 363, 28–37. doi:10.1016/S0140-6736(03)15166-5.

Faideau, M., Kim, J., Cormier, K., Gilmore, R., Welch, M., Auregan, G., et al. (2010). In vivo expression of polyglutamine-expanded huntingtin by mouse striatal astrocytes impairs glutamate transport: A correlation with Huntington’s disease subjects. *Hum. Mol. Genet.* 19, 3053–3067. doi:10.1093/hmg/ddq212.

Ferraiuolo, L., Higginbottom, A., Heath, P. R., Barber, S., Greenald, D., Kirby, J., et al. (2011). Dysregulation of astrocyte-motoneuron cross-talk in mutant superoxide dismutase 1-related amyotrophic lateral sclerosis. *Brain* 134, 2627–41. doi:10.1093/brain/awr193.

Ferraro, T. N., Golden, G. T., Smith, G. G., Martin, J. F., Lohoff, F. W., Gieringer, T. A., et al. (2004). Fine mapping of a seizure susceptibility locus on mouse Chromosome 1: Nomination of Kcnj10 as a causative gene. *Mamm. Genome* 15, 239–51. doi:10.1007/s00335-003-2270-3.

Griciuc, A., Serrano-Pozo, A., Parrado, A. R., Lesinski, A. N., Asselin, C. N., Mullin, K., et al. (2013). Alzheimer’s disease risk gene cd33 inhibits microglial uptake of amyloid beta. *Neuron* 78, 631–43. doi:10.1016/j.neuron.2013.04.014.

Haindl, M. T., Köck, U., Zeitelhofer-Adzemovic, M., Fazekas, F., and Hochmeister, S. (2019). The formation of a glial scar does not prohibit remyelination in an animal model of multiple sclerosis. *Glia* 67, 467–481. doi:10.1002/glia.23556.

Howland, D. S., Liu, J., She, Y., Goad, B., Maragakis, N. J., Kim, B., et al. (2002). Focal loss of the glutamate transporter EAAT2 in a transgenic rat model of SOD1 mutant-mediated amyotrophic lateral sclerosis (ALS). *Proc. Natl. Acad. Sci. U. S. A.* 99, 1604–1609. doi:10.1073/pnas.032539299.

Huang, B., Wei, W. J., Wang, G., Gaertig, M. A., Feng, Y., Wang, W., et al. (2015). Mutant huntingtin downregulates myelin regulatory factor-mediated myelin gene expression and affects mature oligodendrocytes. *Neuron* 85, 1212–1226. doi:10.1016/j.neuron.2015.02.026.

Johann, S., Heitzer, M., Kanagaratnam, M., Goswami, A., Rizo, T., Weis, J., et al. (2015). NLRP3 inflammasome is expressed by astrocytes in the SOD1 mouse model of ALS and in human sporadic ALS patients. *Glia* 63, 2260–2273. doi:10.1002/glia.22891.

Jones, V. C., Atkinson-Dell, R., Verkhratsky, A., and Mohamet, L. (2017). Aberrant iPSC-derived human astrocytes in Alzheimer’s disease. *Cell Death Dis.* 8, 1–11. doi:10.1038/cddis.2017.89.

Lastres-Becker, I., Ulusoy, A., Innamorato, N. G., Sahin, G., Rábano, A., Kirik, D., et al. (2012). α-synuclein expression and Nrf2 deficiency cooperate to aggravate protein aggregation, neuronal death and inflammation in early-stage Parkinson’s disease. *Hum. Mol. Genet.* 21, 3173–3192. doi:10.1093/hmg/dds143.

Lee, T. S., Eid, T., Mane, S., Kim, J. H., Spencer, D. D., Ottersen, O. P., et al. (2004). Aquaporin-4 is increased in the sclerotic hippocampus in human temporal lobe epilepsy. *Acta Neuropathol.* 108, 493–502. doi:10.1007/s00401-004-0910-7.

Lee, Y., Morrison, B. M., Li, Y., Lengacher, S., Farah, M. H., Hoffman, P. N., et al. (2012). Oligodendroglia metabolically support axons and contribute to neurodegeneration. *Nature* 487, 443–448. doi:10.1038/nature11314.

Mason, J. L., Toews, A., Hostettler, J. D., Morell, P., Suzuki, K., Goldman, J. E., et al. (2004). Oligodendrocytes and Progenitors Become Progressively Depleted within Chronically Demyelinated Lesions. *Am. J. Pathol.* 164, 1673–1682. doi:10.1016/S0002-9440(10)63726-1.

Morales, I., Sanchez, A., Rodriguez-Sabate, C., and Rodriguez, M. (2017). Striatal astrocytes engulf dopaminergic debris in Parkinson’s disease: A study in an animal model. *PLoS One* 12, e0185989. doi:10.1371/journal.pone.0185989.

Oksanen, M., Petersen, A. J., Naumenko, N., Puttonen, K., Lehtonen, Š., Gubert Olivé, M., et al. (2017). PSEN1 Mutant iPSC-Derived Model Reveals Severe Astrocyte Pathology in Alzheimer’s Disease. *Stem Cell Reports* 9, 1885–1897. doi:10.1016/j.stemcr.2017.10.016.

Philips, T., Bento-Abreu, A., Nonneman, A., Haeck, W., Staats, K., Geelen, V., et al. (2013). Oligodendrocyte dysfunction in the pathogenesis of amyotrophic lateral sclerosis. *Brain* 136, 471–482. doi:10.1093/brain/aws339.

Politis, M., Lahiri, N., Niccolini, F., Su, P., Wu, K., Giannetti, P., et al. (2015). Increased central microglial activation associated with peripheral cytokine levels in premanifest Huntington’s disease gene carriers. *Neurobiol. Dis.* 83, 115–21. doi:10.1016/j.nbd.2015.08.011.

Robel, S., Buckingham, S. C., Boni, J. L., Campbell, S. L., Danbolt, N. C., Riedemann, T., et al. (2015). Reactive astrogliosis causes the development of spontaneous seizures. *J. Neurosci.* 35, 3330–3345. doi:10.1523/JNEUROSCI.1574-14.2015.

Savage, J. C., St-Pierre, M. K., Carrier, M., El Hajj, H., Novak, S. W., Sanchez, M. G., et al. (2020). Microglial physiological properties and interactions with synapses are altered at presymptomatic stages in a mouse model of Huntington’s disease pathology. *J. Neuroinflammation* 17, 1–18. doi:10.1186/s12974-020-01782-9.

Schofield, E., Kersaitis, C., Shepherd, C. E., Kril, J. J., and Halliday, G. M. (2003). Severity of gliosis in Pick’s disease and frontotemporal lobar degeneration: Tau-positive glia differentiate these disorders. *Brain* 126, 827–840. doi:10.1093/brain/awg085.

Sjögren, M., Folkesson, S., Blennow, K., and Tarkowski, E. (2004). Increased intrathecal inflammatory activity in frontotemporal dementia: Pathophysiological implications. *J. Neurol. Neurosurg. Psychiatry* 75, 1107–11. doi:10.1136/jnnp.2003.019422.

Solano, R. M., Casarejos, M. J., Menéndez-Cuervo, J., Rodriguez-Navarro, J. A., De Yébenes, J. G., and Mena, M. A. (2008). Glial dysfunction in parkin null mice: Effects of aging. *J. Neurosci.* 28, 598–611. doi:10.1523/JNEUROSCI.4609-07.2008.

Stenovec, M., Trkov, S., Lasič, E., Terzieva, S., Kreft, M., Rodríguez Arellano, J. J., et al. (2016). Expression of familial Alzheimer disease presenilin 1 gene attenuates vesicle traffic and reduces peptide secretion in cultured astrocytes devoid of pathologic tissue environment. *Glia* 64, 317–329. doi:10.1002/glia.22931.

Tong, X., Ao, Y., Faas, G. C., Nwaobi, S. E., Xu, J., Haustein, M. D., et al. (2014). Astrocyte Kir4.1 ion channel deficits contribute to neuronal dysfunction in Huntington’s disease model mice. *Nat. Neurosci.* 17, 694–703. doi:10.1038/nn.3691.

Umoh, M. E., Dammer, E. B., Dai, J., Duong, D. M., Lah, J. J., Levey, A. I., et al. (2018). A proteomic network approach across the ALS ‐ FTD disease spectrum resolves clinical phenotypes and genetic vulnerability in human brain. *EMBO Mol. Med.* 10, 48–62. doi:10.15252/emmm.201708202.

Voß, E. V., Škuljec, J., Gudi, V., Skripuletz, T., Pul, R., Trebst, C., et al. (2012). Characterisation of microglia during de- and remyelination: Can they create a repair promoting environment? *Neurobiol. Dis.* 45, 519–528. doi:10.1016/j.nbd.2011.09.008.

Wallraff, A., Köhling, R., Heinemann, U., Theis, M., Willecke, K., and Steinhäuser, C. (2006). The impact of astrocytic gap junctional coupling on potassium buffering in the hippocampus. *J Neurosci.* 26, 5438–47. doi:10.1523/JNEUROSCI.0037-06.2006.

Wilcock, D. M., Vitek, M. P., and Colton, C. A. (2009). Vascular amyloid alters astrocytic water and potassium channels in mouse models and humans with Alzheimer’s disease. *Neuroscience* 159, 1055–1069. doi:10.1016/j.neuroscience.2009.01.023.

Wu, X. L., Zhou, J. S., Wang, L. H., Liu, J. X., Hu, H. B., Zhang, X. T., et al. (2019). Proliferation of NG2 cells in the epileptic hippocampus. *Epilepsy Res.* 152, 62–72. doi:10.1016/j.eplepsyres.2019.03.006.

Zeng, X. N., Sun, X. L., Gao, L., Fan, Y., Ding, J. H., and Hu, G. (2007). Aquaporin-4 deficiency down-regulates glutamate uptake and GLT-1 expression in astrocytes. *Mol. Cell. Neurosci.* 34, 34–39. doi:10.1016/j.mcn.2006.09.008.

Zhao, W., Xie, W., Le, W., Beers, D. R., He, Y., Henkel, J. S., et al. (2004). Activated microglia initiate motor neuron injury by a nitric oxide and glutamate-mediated mechanism. *J. Neuropathol. Exp. Neurol.* 63, 964–977. doi:10.1093/jnen/63.9.964.

Zhao, X., Liao, Y., Morgan, S., Mathur, R., Feustel, P., Mazurkiewicz, J., et al. (2018a). Noninflammatory Changes of Microglia Are Sufficient to Cause Epilepsy. *Cell Rep.* 22, 2080–2093. doi:10.1016/j.celrep.2018.02.004.

Zhao, Y., Wu, X., Li, X., Jiang, L. L., Gui, X., Liu, Y., et al. (2018b). TREM2 Is a Receptor for β-Amyloid that Mediates Microglial Function. *Neuron* 97, 1023-1031.e7. doi:10.1016/j.neuron.2018.01.031.

Zhukareva, V., Mann, D., Pickering-Brown, S., Uryu, K., Shuck, T., Shah, K., et al. (2002). Sporadic Pick’s disease: A tauopathy characterized by a spectrum of pathological τ isoforms in gray and white matter. *Ann. Neurol.* 51, 730–739. doi:10.1002/ana.10222.
